# Machine Learning as a Tool for Early Detection: A Focus on Late-Stage Colorectal Cancer across Socioeconomic Spectrums

**DOI:** 10.3390/cancers16030540

**Published:** 2024-01-26

**Authors:** Hadiza Galadima, Rexford Anson-Dwamena, Ashley Johnson, Ghalib Bello, Georges Adunlin, James Blando

**Affiliations:** 1 School of Community and Environmental Health, Old Dominion University, Norfolk, VA 23529, USA; ranso002@odu.edu (R.A.-D.); ajohn239@odu.edu (A.J.); jblando@odu.edu (J.B.); 2 Department of Environmental Medicine & Public Health, Icahn School of Medicine at Mount Sinai, New York, NY 10029, USA; ghalibbello@gmail.com; 3 Department of Pharmaceutical, Social and Administrative Sciences, Samford University, Birmingham, AL 35229, USA; gadunlin@samford.edu

**Keywords:** socioeconomic disparity, cancer care, predictive modeling, machine learning, AI, social determinants of health in oncology, spatial analysis, precision care

## Abstract

**Simple Summary:**

This research explores the potential of machine learning (ML) to predict late-stage colorectal cancer (CRC) diagnoses. The focus is on understanding how socioeconomic and regional factors affect cancer care, particularly in detecting CRC at an advanced stage. We aim to merge data on social determinants of health with individual demographics to uncover patterns indicating higher CRC risk. We compared various ML models, such as decision trees, random forest, and gradient boosting to find the most effective tool for this task. The goal is to utilize artificial intelligence (AI) for early, more accurate CRC detection, which can lead to better treatment outcomes. This study promises to significantly contribute to cancer research, potentially leading to more personalized and efficient healthcare strategies that could ultimately save lives.

**Abstract:**

Purpose: To assess the efficacy of various machine learning (ML) algorithms in predicting late-stage colorectal cancer (CRC) diagnoses against the backdrop of socio-economic and regional healthcare disparities. Methods: An innovative theoretical framework was developed to integrate individual- and census tract-level social determinants of health (SDOH) with sociodemographic factors. A comparative analysis of the ML models was conducted using key performance metrics such as AUC-ROC to evaluate their predictive accuracy. Spatio-temporal analysis was used to identify disparities in late-stage CRC diagnosis probabilities. Results: Gradient boosting emerged as the superior model, with the top predictors for late-stage CRC diagnosis being anatomic site, year of diagnosis, age, proximity to superfund sites, and primary payer. Spatio-temporal clusters highlighted geographic areas with a statistically significant high probability of late-stage diagnoses, emphasizing the need for targeted healthcare interventions. Conclusions: This research underlines the potential of ML in enhancing the prognostic predictions in oncology, particularly in CRC. The gradient boosting model, with its robust performance, holds promise for deployment in healthcare systems to aid early detection and formulate localized cancer prevention strategies. The study’s methodology demonstrates a significant step toward utilizing AI in public health to mitigate disparities and improve cancer care outcomes.

## 1. Introduction

Colorectal cancer (CRC) represents one of the most prevalent malignancies globally [1], standing as the third most commonly diagnosed cancer in both men and women and the third leading cause of cancer-related deaths in the United States in 2023 [2]. Overall, the incidence of CRC is declining among adults in the U.S., influenced by multiple factors. Increased adherence to CRC screening plays a crucial role in early detection, resulting in higher curability rates and improved survival rates [2,3,4,5,6,7]. Additionally, factors such as dietary improvements and lifestyle changes also contribute to lower incidence by preventing cancer occurrence [8,9]. However, despite advancements in detection methods and treatment modalities to reduce the overall incidence of advanced-stage CRC, it remains a significant public health challenge owing to its substantial mortality rates [2,10]. Comprehensive understanding and awareness of its risk factors, prevention strategies, and early symptoms are essential in combating its progression and ensuring timely medical intervention.

Literature has recognized a combination of genetic, environmental, and lifestyle factors plays a contributory role in CRC etiology [11]. Several demographic variables are related to CRC health outcomes, disparity, and mortality, including sex, age, race/ethnicity, and others. These demographic variables have also been proposed as factors influencing the determination of disease progression at diagnosis, ultimately impacting treatment decisions and plans of care. Several studies have also highlighted the lifelong effects on health that social determinants of health (SDOHs)—defined as the “conditions in which people are born, grow, live, work, and age” [12]—and their interactions with non-modifiable factors can have [13,14]. However, such studies tend to focus on elucidating the effect-modifying role of one factor at a time, even though other factors may be involved. A reason for this approach may be the scarcity of analytical tools (due to analytical complexity) for properly accounting for the complex interaction patterns that exist among several potential cancer factors. A more comprehensive approach to describing and evaluating how multi-level factors affect cancer health outcomes require a clear emphasis on how multiple factors at different levels interact with one another. One promising approach to this problem is machine learning. While there are well-established ‘traditional’ statistical methods to predict cancer-risk outcomes based on several potential risk factors, many of these methods are not optimal for accurately delineating complex non-linear relationships that might exist between many risk factors and an outcome of interest. Machine learning (ML) techniques allow for an agnostic, data-driven approach to map out potentially complex relationships that are difficult to specify analytically (e.g., in the form of a regression-type equation).

### 1.1. Machine Learning Approaches in Predicting Cancer Outcomes—A Literature Review

Machine learning (ML) is a rapidly evolving field within biomedical research, particularly in oncology. It leverages the vast amount of data collected from various health platforms to enhance the precision of cancer diagnoses [15,16]. At its core, ML involves the use of algorithms and statistical techniques to analyze and learn from data patterns [17,18,19]. In the context of cancer research, ML techniques often utilize rich gene expression datasets and integrate various risk factors to create predictive models [20,21,22]. For instance, in a study by Hornbrook et al, ML integrated demographic factors such as gender and age with clinical metrics, such as blood count to predict early colorectal cancer [23]. Following a similar trajectory, numerous studies have successfully employed ML to integrate clinical parameters such as tumor grade, blood indices, gender, smoking history, and age, leading to accurate predictions of bone metastases in thyroid cancer patients [24]. Similar factors have been instrumental in predicting the probability of advanced colorectal neoplasia in asymptomatic adults using deep learning model [25]. There is a growing repository of ML models constructed on data sourced from cancer databases such as the surveillance, epidemiology, and end results (SEER) Program (https://seer.cancer.gov/), which utilize predictors encompassing tumor attributes, demographic characteristics, and clinical characteristics, to forecast metrics such as mortality and survivorship across a range of cancers including oral, endometrial, and lung cancers [26,27,28,29,30].

Recent advancements in ML, particularly methods such as artificial neural networks (ANN) and deep learning, have showcased promising results. They have been pivotal in estimating the likelihood of cancers such as lung and colorectal spreading further. These techniques integrate both clinical and demographic data for their predictions [31,32]. Moreover, ML models have been meticulously curated to classify patients based on their susceptibility to cancer development, disease stage, and potential treatment outcomes, employing data from varied sources such as electronic health record (EHR) and SEER [33,34,35].

Furthermore, certain studies have explored the complex interplay between different variables using ML techniques. A prime example would be the study by Levitsky et al., which employed patient-reported data within an ML framework, aiming to discern how varying factors might collaboratively influence the prediction of lung cancer onset [36]. In summation, machine learning, with its innovative approaches, emerges as an invaluable tool in contemporary medical research that could significantly enhance conventional methods of predicting cancer outcomes.

### 1.2. Role of Social Determinants of Health in Cancer Research

In line with population and public health research, increasingly, social determinants of health (SDOHs), at the time of diagnosis or study enrollment are collected in epidemiologic studies of cancer that enable a comprehensive analysis of cancer risk factors [37,38,39,40]. While population-based cancer registries play a crucial role in monitoring cancer trends, they may not comprehensively capture all incidences of cancer. Additionally, limitations in the collection of variables for hypothesis-driven research within these registries can potentially introduce bias into the results [41,42,43]. For example, information on patient insurance status, patient comorbidities, and active follow-up of patients are inconsistently available. Cancer data can be improved when used in combination with other secondary databases containing social determinants of health to increase our understanding of the underlying causes of cancer health disparities [43,44].

The influence of SDOH, neighborhood and environmental attributes on cancer-related outcomes such as mortality, survivorship, and stage at diagnosis has been increasingly recognized in the scientific community. Health care accessibility, represented by factors such as the presence or absence of a primary care physician and insurance status, holds substantial sway over cancer screening rates and subsequent outcomes. For instance, residing in neighborhoods characterized by high levels of racial and ethnic segregation has been associated with low cancer screening rates and detrimental health outcomes [45,46]. Notably, a lack of insurance coverage has been linked to poorer health outcomes [47,48,49,50,51,52]. Socioeconomic elements, including financial insecurity, poverty, low-income status, and employment conditions, also exert a significant impact on cancer-related health metrics [45,48,52,53,54]. In addition, adverse social and educational determinants such as high social vulnerability and limited educational attainment have been correlated with negative outcomes in cancer screening and overall health [45,55].

In addition to these established factors, other variables have emerged as potentially relevant based on authoritative recommendations. Korn et al. proposed a systematic review protocol to compile variables related to SDOH constructs for cancer screening and outcomes. These include variables such as food insecurity, housing, language and literacy skills, transportation, affordability, incarceration, and more [56]. These SDOH constructs were identified and organized based on established frameworks and definitions, providing a comprehensive view of the complex interplay between SDOH and cancer outcomes.

Through these studies, it becomes evident that SDOH are not merely peripheral elements but integral components that significantly modulate cancer outcomes. This evidence emphasizes the need for a more holistic approach to cancer research and treatment, one that encompasses the complex interplay of these determinants.

### 1.3. Study Purpose

The purpose of this study is to explore the advanced capabilities of machine learning (ML) and artificial intelligence (AI) in predicting late-stage colorectal cancer (CRC) diagnoses, within the broader context of addressing socioeconomic and regional disparities in cancer care. By integrating individual- and census tract-level social determinants of health (SDOHs) with sociodemographic factors, the study aims to identify patterns that may signal a higher risk of late-stage CRC, which is characterized here as diagnoses made at regional stage or with distant metastasis/systemic disease. Central to the research is the comparative analysis of a suite of ML models—including penalized lasso logistic regression, decision tree, random forest, gradient boosting, and SVM classifier—to evaluate their predictive prowess and ensure the most effective algorithm is deployed for this critical healthcare challenge. This approach, which focuses on prognostic predictions using ML algorithms, is a crucial element in cancer care, offering potential for early detection and improved treatment outcomes. The study’s ultimate goal is to harness the power of AI to generate precise, population-level predictions, thereby contributing significantly to the field of cancer care and management.

## 2. Materials and Methods

### 2.1. Study Design and Setting

This study uses an ecological design to examine CRC outcomes for the period 2000 to 2020 in men and women aged 18 years of age and older residing in the state of Virginia. The chosen study setting offers a distinct structure for exploring CRC since it represents a significant health issue in Virginia, where 26 counties have been recognized as having the highest colorectal cancer mortality rates in the country [57,58,59].

### 2.2. Theoretical Framework

**Figure 1:**
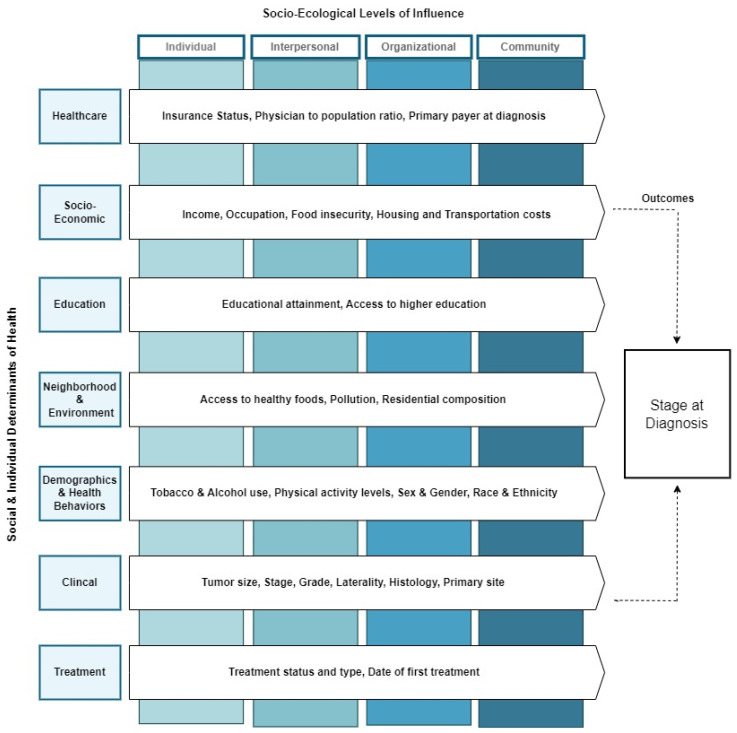
Adaptive predictive framework for cancer outcomes (APF-CO).

This study introduces an innovative theoretical framework, the adaptive predictive framework for cancer outcomes (APF-CO) in Figure 1, that synergistically merges elements from the social-ecological model [60], the healthy people 2030 model [61], and the Kaiser Foundation’s framework on social determinants of health (SDOHs) [62]. Designed to foster a nuanced understanding of cancer outcomes, this integrative framework serves as a substrate for employing machine learning algorithms to predict cancer outcomes effectively.

In this multifaceted approach, we combine both clinical variables and SDOH to construct a more holistic understanding of cancer outcomes. The framework is structured around several key categories that span individual and societal determinants of health: healthcare access, socio-economic status, diet and physical activity, educational attainment, environmental factors, neighborhood characteristics, social and community support, clinical metrics, treatment options, demographic attributes, and health behaviors.

To add depth and context to our framework, these categories are strategically mapped across four levels of influence as outlined in the social-ecological model: individual, interpersonal, organizational, and community. The intention behind this mapping is to illustrate that variables from these categories are not isolated entities but interact dynamically across different layers of the social fabric. This facilitates a comprehensive understanding of how such complex interactions can exert a cascading influence on cancer outcomes.

By amalgamating a wealth of variables, drawn from diverse yet interconnected domains, this modified theoretical framework offers an enriched, layered perspective. The ultimate goal is to capitalize on machine-learning methodologies to decipher the intricate web of determinants, thereby offering predictive understandings that are both accurate and encompassing. This unprecedented approach, incorporating an expansive database that combines traditional cancer registry data with metrics related to SDOH, sets the stage for ground-breaking advancements in predictive oncology.

### 2.3. Data Sources

The primary data for this project i.e., cancer cases specific to CRC are obtained from the Virginia Cancer Registry (VCR) [63]. The data include information on patient demographic characteristics, residential locations, as well as histological and clinical characteristics, among other information. Census tracts-level social determinants of health include data from the U.S. Census Bureau’s 2019 American Community Survey 5-year estimates (2015–2019) [64], and from the mySidewalk health data [65]. mySidewalk includes more than 6000 preloaded community-level indicators from more than 50 trusted sources for the entire country; these include data from the National Neighborhood Data Archive (NaNDA), the Health Resources and Services Administration (HRSA), the Centers for Disease Control (CDC), the Environmental Protection Agency (EPA) National Air Toxics Assessment, etc. [65]. These datasets are merged with the VCR dataset based on the patient’s residential census tract at time of diagnosis as a common identifier.

### 2.4. Study Population

Individuals aged 18 to 89, residing in Virginia between 2000 and 2020, and who were diagnosed with primary and secondary CRC were selected for the analysis. The CRC cases were identified using the following International Classification of Disease, 10th revision, Clinical Modification (ICD-10-CM) codes: C18.0-C18.9, C19.9, and C20. Cases with missing or invalid residential information at diagnosis were excluded.

### 2.5. Data Collection and Variables Definitions

Individual-level Variables: Baseline characteristics were obtained from the VCR. They include patient demographic characteristics (age at diagnosis, sex, patient race and ethnicity, marital status), year of CRC diagnosis, primary payer at diagnosis, residential location (county, zip code, census tract); clinical characteristics such as tumor stage at the time of diagnosis, tumor characteristics, primary tumor site, histologic type, grade, laterality, and treatment information. Previous studies have shown individual factors that have been associated with adverse cancer outcomes include age, gender, race and ethnicity [66,67]. Important individual clinical factors include tumor size and thickness, tumor grade, histology, stage at diagnosis, lymph node count and location, and treatment types [68,69,70,71].

Neighborhood Social Determinant of Health Measures: The neighborhood SDOH variables used in this study are summarized in Figure 1. They include socioeconomic, racial, and geographic disparities (urban vs. rural). It has been shown that there is a higher incidence and risk of adverse CRC outcomes amongst rural populations when compared to urban populations [52,72,73].

Outcome Measure: The primary outcome of interest is a binary variable representing the stage of CRC at diagnosis (late-stage vs. early stage). We defined “late” stage as all CRC diagnoses recorded at regional stage (regional by direct extension, regional lymph nodes only, and regional by both extension and lymph nodes) and distant metastasis/systemic disease. We defined ‘early’ stage as all CRC diagnoses recorded as ‘localized’ and in situ [70].

### 2.6. Data Linkage and Management

The integration of the cancer registry data and the social determinants of health (SDOH) information from census tracts was pivotal in constructing a comprehensive dataset that not only highlights individual cancer outcomes but also provides an invaluable lens into their potential interplays with socio-economic determinants at the micro-geographic level. The primary dataset from the cancer registry contained specific residence information for each patient. These residence data served as a key attribute for the data linkage.

Initially, we accessed a comprehensive dataset from a 20-year-long cancer registry, revealing 74,921 documented CRC cases. Using the ArcGIS platform, a majority of the cancer cases were geocoded based on patients’ residence information, translating them to specific geographic co-ordinates. As a result, 74,435 records (99.35% of the total cases) were perfectly georeferenced into their respective census tracts. For the 0.65% (486 records) that were not initially georeferenced, the Missouri Census Data Center’s (MCDC) “apportioned” population weight methodology was employed. This method, available on the MCDC’s online platform [74], employs patients’ zip codes to transition or “crosswalk” these cases into designated census tracts. The procedure involves assigning a case to the census tract with the highest population percentage from the given zip code, resulting in the zip code-census tract crosswalk methodology. From the 486 records processed using MCDC, 454 were successfully matched, and only 32 records (0.04% of the total sample) remained un-georeferenced.

The culmination of the linkage process yielded a unified dataset, encompassing 87 features and a total of 74,921 rows. Rigorous data management protocols were meticulously applied post-integration to address any missing values, discrepancies, or inconsistencies that might have emerged during the merger. Specifically, an initial assessment revealed that 75 features had missing values. To ensure data quality, columns with more than 90% missing values were dropped, and missing values in numerical features were imputed with median values, ensuring minimal impact on data distribution, while categorical features were filled with a placeholder string ‘unknown’, preserving the original structure without introducing bias. Rows lacking the crucial ‘stage at diagnosis’ data were also removed, resulting in a refined dataset of 41,839 samples and 86 features. This rigorous approach enhanced the dataset’s analytical robustness and reliability.

### 2.7. An Overview of Machine Learning Techniques in Predicting Cancer Outcomes

Lasso Logistic Regression, a variant of logistic regression, is a statistical method used for modeling the probability of a binary outcome based on multiple predictor variables. This method, ideal for handling high-dimensional data, simplifies the model by reducing the coefficients of less significant predictors. More specifically, this method reduces the impact of less significant variables by shrinking their coefficients towards zero, thus simplifying the model and enhancing interpretability [75,76,77].

Decision trees is a machine learning algorithm used for both classification and regression tasks. This algorithm operates by sorting instances based on their feature values through a process known as recursive binary partitioning. Each node in a decision tree represents a specific feature, and each branch represents a decision path based on feature values that leads to the next node [78,79]. They function through a sequence of binary decisions: for example, in our context of 86 features, if a feature value $X_{k}$ is less than or equal to threshold S, the tree follows the left branch; otherwise, it follows the right. This process continues recursively until a leaf node, representing the final decision, is reached. The process of node selection and thresholds determination (‘S’) is determined through optimization techniques, making decision trees highly adaptable to specific datasets.

Random forest is an ensemble learning method. It operates by constructing a multitude of decision trees during the training phase. For classification tasks, the random forest model predicts the output class by employing a majority voting system across all the decision trees, where each tree votes for a class, and the class receiving the most votes becomes the model’s prediction. For regression tasks, it aggregates the predictions of individual trees by calculating their mean. Unlike a single decision tree, random forest reduces the risk of overfitting and increases prediction accuracy by combining the predictions of numerous trees [79,80].

Gradient Boosting is a powerful machine learning technique used in both regression and classification problems. It operates by training decision trees iteratively [81]. Each subsequent tree in a gradient-boosted model focuses more on observations that previous trees predicted inaccurately, essentially placing higher weight on these errors. This error-correcting approach allows gradient boosting to continuously improve its predictions throughout the training process [79]. The emphasis in gradient boosting is on improving accuracy and reducing bias through iterative refinement, making it particularly effective in complex predictive tasks such as cancer diagnosis and prognosis.

Support Vector Machines (SVMs) are supervised learning models that analyze data and recognize patterns. They are used for classification and regression analysis. In scenarios where data are linearly separable, SVMs employ a linear approach to establish a hyperplane that best divides the data into distinct classes [82]. However, given the complexity and non-linear relationships often present in medical datasets, including those related to cancer, the use of SVMs with non-linear kernels is recommended. These non-linear kernels enable the delineation of intricate decision boundaries and are more adept at handling high-dimensional data.

### 2.8. Statistical Analyses

The cancer registry and population census data were merged to integrate records from both sources. Patients’ characteristics between early- and late-stage diagnosis were compared in a bivariate analysis using chi-square tests for categorical variables and *t*-tests for continuous variables. Similarly, *t*-tests were utilized to compare the SDOH characteristics at the census tract levels.

In the exploratory data analysis phase, several machine learning models from the scikit-learn library to predict late-stage cancer diagnosis were used. This array included lasso logistic regression, employing L1 regularization with the ‘saga’ solver; decision tree; random forest; gradient boosting; and a variant of support vector machine (SVM), specifically the SGD (stochastic gradient descent) classifier with hinge loss function. Notably, this method serves as a computationally efficient approximation of SVMs, leveraging stochastic gradient descent for optimization in high-volume data scenarios. The random forest model was configured with its default number of tree estimators, while the gradient boosting classifier utilized Scikit-learn’s default settings. These default hyperparameter settings were chosen for an initial broad assessment. Each model was configured to address class imbalance using class weights. 

To minimize overfitting and rigorously evaluate the performance of the models, a 5-fold cross-validation approach was used. This approach involved splitting the data into five distinct subsets or ‘folds’. In each iteration, four folds were used for training while the remaining fold served as the validation set. This process was repeated five times, ensuring each fold was used for validation once. By doing so, this method offers a more comprehensive assessment of a model’s performance across different data subsets, reducing biases and providing an indication of its generalization capabilities. Performance metrics including ROC-AUC, accuracy, sensitivity, and specificity were computed, capturing different aspects of model efficacy. Additionally, for a comprehensive geographical analysis, the best-performing model, based on these metrics, was used to estimate late-stage cancer probabilities across various census tracts in Virginia, employing spatial–temporal analysis for visualizing and identifying significant clusters over time.

All data management and spatial analyses were conducted using SAS and ArcGIS, respectively, with Python and Scikit-learn serving as the primary tools for machine learning model fitting and evaluation. The level of significance was set at *p* < 0.05, ensuring statistical rigor in our findings.

## 3. Results

### 3.1. Baseline Characteristics

Baseline characteristics between early-stage ((42.74%) and late-stage (57.26%) diagnosis are summarized in Table 1. Patients diagnosed at an early stage had a slightly higher mean age (66.72 years) than those diagnosed at a late stage (65.85 years, *p* < 0.0001). A significantly greater proportion of patients aged 40–49 were diagnosed at late stage (*p* < 0.0001). Concerning race and ethnicity, there were slight variations, with Hispanics and NH Asians being marginally overrepresented in the late diagnosis group (*p* = 0.005).

Marital status significantly affected the stage at diagnosis. Married individuals were more frequently diagnosed early, while a larger percentage of unmarried patients were diagnosed at late stage (*p* < 0.0001). The primary payer at diagnosis showed that Medicaid beneficiaries and the self-pay/uninsured group were more prone to late stage (*p* < 0.0001). The year of diagnosis revealed temporal differences in staging. For instance, the period between 2015 and 2019 saw a surge in late diagnoses (*p* < 0.0001). The stage of the disease at diagnosis was as expected, with in situ and localized stages in the early diagnosis group, contrasted by regional and distant stages in the late group.

Primary site analysis showed that colon diagnoses were more predominant in the late diagnosis group, whereas rectum diagnoses leaned towards the early diagnosis group (*p* < 0.0001). Regarding disease grade, early diagnoses had a greater proportion of Grade I, while late diagnoses were rich in Grade II and III cases (*p* < 0.0001).

Treatment patterns displayed notable differences between the groups. Surgery was more common in the early diagnosis group, whereas chemotherapy and radiation therapy were more prevalent in the late diagnosis group (*p* < 0.0001). The vital status highlighted a concerning trend, with a majority (62.03%) of the late diagnosis group succumbing to the disease, in comparison to 40.68% in the early diagnosis group (*p* < 0.0001). Detailed information on the baseline characteristics can be found in the Supplementary Content in Table S1: Baseline Characteristics by Stage of Diagnosis Status.

### 3.2. Census Tracts Characteristics by Stage at Diagnosis

The bivariate analysis of census tract SDOH characteristics and stage at diagnosis, displayed in Table 2, reveals some intriguing disparities between early- (42.74%) and late-stage (57.26%) patients. Access to healthcare, socio-economic status, education, lifestyle behaviors, ethnicity, and environmental exposure all display certain associations with the stage of diagnosis. Key differences include a higher percentages of uninsured individuals, less routine check-ups, and lower access to healthy food in tracts with late-stage patients. Socio-economic factors such as lower household income, higher expenditure on transportation, and less workforce participation also correlate with late-stage diagnoses. Educationally, those areas tend to have more people with less than high school education, but fewer with some college education but no degree. Interestingly, areas with late-stage diagnoses also recorded higher levels of binge drinking and a higher Hispanic population percentage. Environmental exposure factors, including slightly elevated diesel particulate matter levels and more prevalent underground storage tanks, were also more common in areas with late-stage diagnoses. These findings suggest that a range of socioeconomic, behavioral, and environmental factors may influence the stage at which patients are diagnosed with cancer. Detailed information on the census tracts characteristics can be found in the Supplementary Content in Table S2: Neighborhood Census Tracts Characteristics by Stage of Diagnosis Status. 

### 3.3. Results of the Predictive Machine Learning Models

Table 3 presents the performance metrics from the cross-validation of five different machine-learning models to predict CRC late-stage diagnosis. In the comparative analysis of the ML models, the gradient boosting model exhibited superior performance with the highest ROC-AUC score of 0.8549, indicating a strong capability in distinguishing between late-stage and non-late-stage CRC diagnoses. It also achieved the highest prediction accuracy at 77.25%, suggesting it is the most reliable model for correct predictions among those tested. In terms of sensitivity, which measures the correct identification of actual late-stage CRC cases, the lasso logistic regression model was the most proficient, with a sensitivity score of 0.7405.

The random forest model, despite its highest specificity (80.72%), fell short in sensitivity (56.40%), indicating a tendency to miss a significant number of late-stage cases. This could be a critical drawback in medical diagnostics where failing to identify late-stage disease could have dire consequences. On the other hand, the decision tree and SVM (SGD classifier) models showed a balance between sensitivity and specificity but lagged in overall accuracy and exhibited low ROC-AUC scores of 0.7298 and 0.7006, respectively, suggesting they might not be as effective for this particular diagnostic challenge.

In conclusion, the gradient boosting stands out as the most promising model, offering a particularly robust approach for this prediction task.

### 3.4. Performance of the Best ML Predictive Model

**Figure 2:**
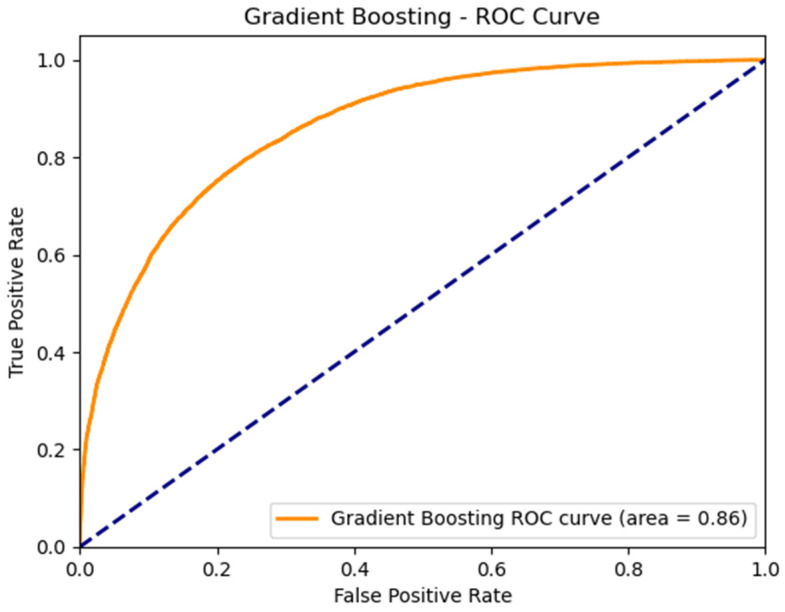
ROC curve from the GBM *. * GBM: Gradient Boosting Model.

**Figure 3:**
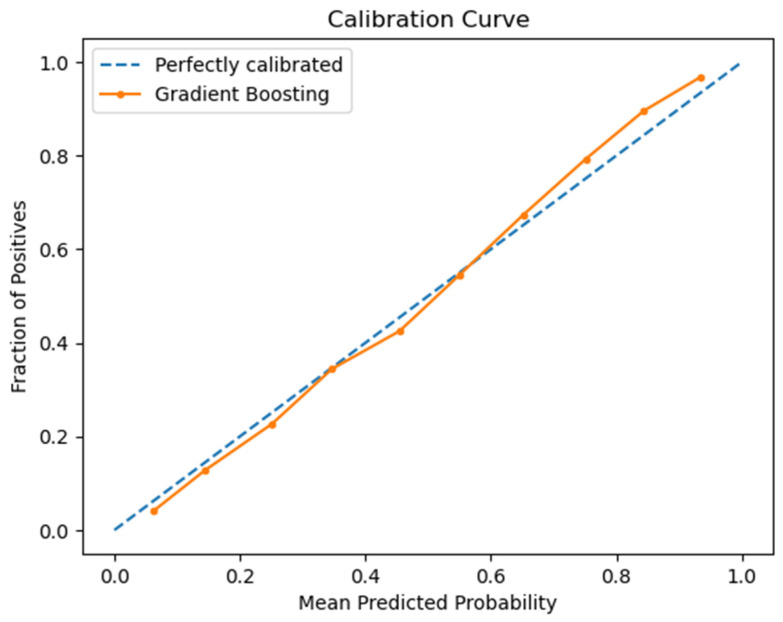
Calibration curve from the GBM *. * GBM: Gradient Boosting Model.

To validate the performance of our gradient boosting model (GBM), which has proven to be the most performant in predicting late-stage colorectal cancer, we conducted a single-run evaluation. This involved partitioning our dataset into an 80–20 split, where 20% constituted the testing set. The model was recalibrated on the training set, then we applied it to the test set to compute critical metrics. The Receiver Operating Characteristic (ROC) curve, depicted in Figure 2, was generated, resulting in an Area Under Curve (AUC) of 0.86—a strong indicator of the model’s discriminative power. To refine the model’s predictive precision, an optimal decision threshold was calculated at 0.446, which improved the model’s sensitivity to 79.54% and specificity to 75.92%, thereby balancing the trade-off between detecting actual cases and avoiding false alarms. Furthermore, the calibration curve, illustrated in Figure 3, was derived from this evaluation, showcasing a close alignment between predicted probabilities and actual outcomes, further confirming the model’s reliability. These measures collectively confirm the robustness of the gradient boosting model as a reliable tool for clinical prognostic applications.

### 3.5. Feature Importance

**Figure 4:**
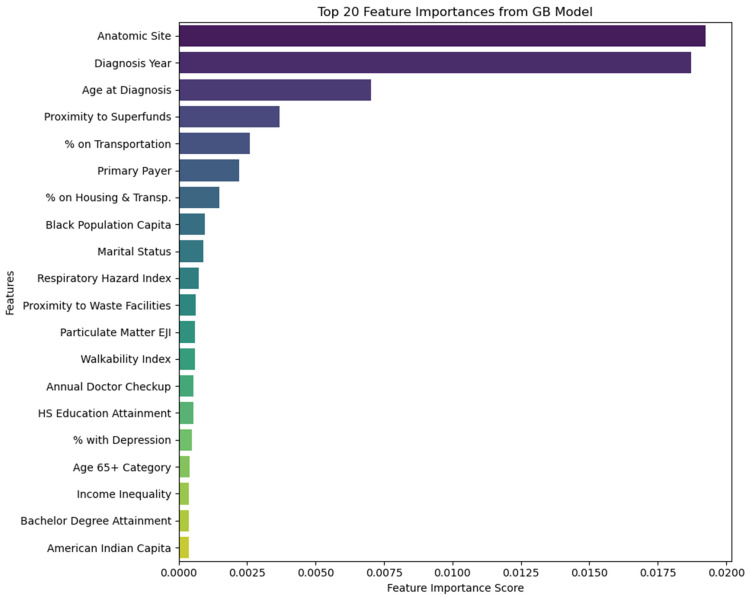
Feature importance for the gradient boosting model.

The feature importance scores from the gradient boosting model, displayed in Figure 4, are indicative of the relative contribution of each predictor to the model’s performance for making predictions. These scores are calculated based on how much each feature contributes to reducing the model’s loss function across all the trees in the ensemble. Each feature’s score is a sum of the reduction in the loss attributed to that feature across all trees in the model.

The anatomic site exhibits the highest importance with an importance score of 0.019238. This signifies that the location of the cancer within the body significantly impacts the stage at diagnosis. The year of diagnosis follows with an importance score of 0.018704, reflecting how advancements or changes in diagnostic methods over time may affect late-stage diagnosis rates. Notably, specific years, such as 2015 and 2016, have been highlighted due to an increased demand for screening [83,84] and the potential delays in diagnosis [85] which can impact the stage at which CRC is identified. Age at diagnosis is also a crucial factor, with an importance score of 0.007015. It is worth noting that age has been established as a significant factor influencing the stage at which CRC is diagnosed [54,70]. Environmental factors such as proximity to superfund sites, though less impactful, still provide meaningful predictive power, suggesting a potential link between environmental factors and disease progression. Socioeconomic factors such as expenditures on transportation and housing, along with the primary payer, are also amongst the top predictors, hinting at the socioeconomic dimensions of healthcare access and outcomes. Social factors such as marital status may reflect the impact of support networks on health, while ‘black per capita’ indicates the potential influence of racial factors and disparities on late-stage diagnosis. Environmental quality indices, including respiratory hazard index and proximity to pollution and waste facilities, alongside health behaviors such as regular checkups and mental health status represented by the percentage with depression, show lower but non-negligible influence. Overall, it is important to note that these importance scores are not indicative of causation or statistical significance. These scores assist in emphasizing the multifaceted nature of cancer diagnosis stages, influenced by a blend of medical, environmental, socioeconomic, and behavioral factors.

### 3.6. Results of the Spatio-Temporal Analysis

**Figure 5:**
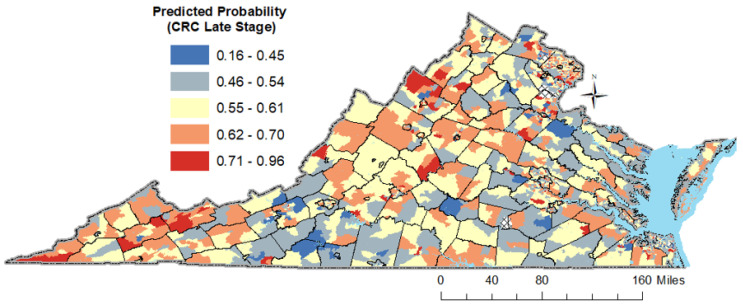
Predicted probability for CRC late stage in Virginia by census tract.

**Figure 6:**
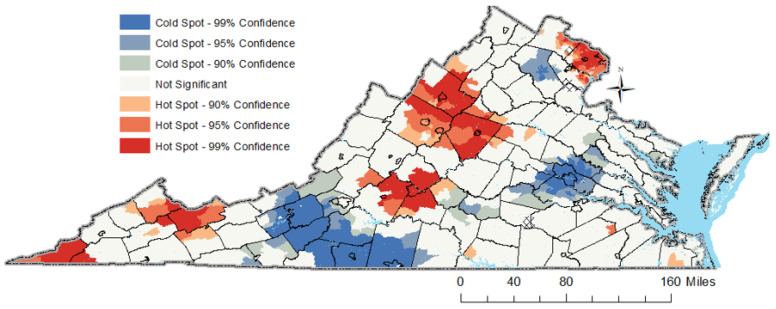
Cluster of high and low predicted probability for CRC late stage.

A preliminary examination of the spatial distribution of anticipated probabilities at the census tract level as displayed in Figure 5, indicates that the greatest likelihood for late stage is observed inside a rural crescent spanning from the Appalachia Mountains to southwest Virginia and in northern Virginia. The analysis presented in Figure 6 highlights several hotspots of late-stage CRC diagnosis across Virginia. Deep red tracts in the southwestern counties, including Lee, Scott, Buchanan, and Tazewell, are predominantly within rural settings and are marked by significant challenges related to socio-economic and healthcare access, potentially contributing to delayed CRC diagnoses. Historically, these areas were heavily reliant on coal mining, which served as a major source of employment and played a significant role in mitigating poverty. However, the decline of the coal industry has not only led to economic challenges but also left a legacy of health exposures for former coal miners. In the western and central regions of Virginia, counties such as Rockingham, Augusta, Albemarle, Amherst, Bedford, and Campbell, exhibit hotspots despite their mixed rural–suburban landscapes and generally favorable socio-economic conditions and healthcare access. Notably, these counties are also characterized by a significant retiree population, suggesting that the age demographic, with a larger proportion of elderly residents, could be influencing the incidence of CRC. Interestingly, northern Virginia, represented by Fairfax County, also emerges as a hotspot highlighting the multifaceted nature of healthcare access and utilization. This county, while boasting a higher socio-economic status (SES), has its own set of challenges that come with affluence, such as increased traffic and resultant air quality concerns. The presence of hotspots across varied socio-economic landscapes underscores the complex interrelation of factors influencing late-stage CRC diagnoses and emphasizes the need for region-specific interventions.

## 4. Discussion

In this work, we presented a ML framework for predicting CRC outcomes. More specifically, the study incorporates an ML model along with spatial accessibility measurement to highlight the spatial disparities in the probability of a late-stage CRC diagnosis based on an array of social determinants of health variables, drawn from diverse yet interconnected domains. The study also introduced an innovative theoretical framework, integrating a wealth of SDOH variables to cancer registry data with a primary emphasis on its generalizability and potential for reuse to predict cancer outcomes using ML techniques. After rigorous data preprocessing, we evaluated the performance of five distinct machine-learning algorithms using the ROC-AUC and other metrics. Notably, the gradient boosting model achieved a superior ability to predict late-stage diagnosis. In contrast to previous studies that predominantly focused on validating machine-learning techniques for predicting cancer diagnoses within the general population, our research extends beyond the validation of the gradient boosting model. We specifically targeted high-risk demographic cohorts and conducted an exhaustive analysis of social determinants of health.

According to our gradient boosting model, the top five contributors to the diagnosis of late-stage CRC, ranked in order of importance, are anatomic site, year of diagnosis, age at diagnosis, proximity to superfund sites, and payer at the time of diagnosis. These findings offer a novel perspective for examining the hierarchy of significance among contributors to late-stage CRC diagnosis when utilizing national data. At a more comprehensive level, encompassing all 20 contributors pinpointed by the gradient boosting model, the factors contributing to the prediction of late-stage CRC diagnosis is ascribed to the intricate interplay among individual-level, community-level, and environmental factors. These results align with established literature, where each of these contributors has previously been recognized as a potential risk factor for late-stage CRC diagnosis [54,86,87]. It goes without saying that the gradient boosting model accurately discerned the key drivers of late-stage CRC diagnosis, marking the initial crucial phase in the development of targeted intervention strategies to enhance outcomes in CRC.

The gradient boosting model has become increasingly instrumental in oncology, offering a multifaceted approach to cancer research, diagnosis, treatment, and prognosis. This powerful algorithm leverages ensemble learning to enhance the accuracy of predictive models and has proven particularly valuable in various facets of cancer management. Gradient boosting finds notable applications in predicting cancer risk, [88] survival, [89,90] and diagnosis stages, as well as assisting in cancer classification [91,92]. The capacity of the gradient boosting model to integrate diverse data sources and discern intricate patterns has proven transformative in advancing the understanding of cancer and improving patient outcomes.

Historical space–time analysis of cancer data has typically entailed examining aggregated data to detect patterns in order to understand how areas with varying levels of risk evolve over time [93,94]. In this study, we also identified spatio-temporal clusters of high and low predicted probability for late-stage CRC. Using the census tract as the unit, the analyses identified about 10 statistically significant clusters in Virginia. The spatio-temporal analysis facilitated the refinement of estimates related to area-level factors and established risk factors for CRC, including their interactive dynamics. Furthermore, this analysis yielded tangible, location-specific evidence that can guide targeted intervention strategies.

## 5. Limitations

Since the gradient boosting model employed in this study adopts a data-driven approach, the model’s performance and predictive capabilities are contingent upon the availability and quality of the data. For example, previous literature has identified several SDOHs that have been associated with poor CRC health outcomes that were unable to be obtained. Some of these include social isolation, neighborhood disadvantage/deprivation, health literacy, and transportation options [45,54,95,96]. Data were analyzed from a single state cancer registry. To further determine and verify the robustness and broad applicability of the results, national cancer databases such as the surveillance, epidemiology, and end results should be analyzed. Regarding the aforementioned limitations, we intend to further investigate them in subsequent studies. Finally, in these analyses default hyperparameter settings were chosen from Scikit-learn for an initial broad assessment, with future work planned to fine-tune these parameters using techniques such as GridSearchCV or RandomizedSearchCV for optimized performance.

## 6. Conclusions

In conclusion, this study has not only showcased the advanced capabilities of machine learning algorithms in predicting late-stage colorectal cancer but also underscored the critical role of spatial accessibility measurements in understanding the disparities in late-stage CRC diagnosis. The spatio-temporal analysis implemented here is particularly instrumental, revealing statistically significant clusters of late-stage CRC diagnoses that necessitate focused public health strategies. These strategies are vital in addressing the observed disparities, reducing the incidence of late-stage CRC, and moving towards the eradication of health inequities. The study’s multifaceted ML approach, with gradient boosting leading the way, has not only validated its predictive accuracy but also illuminated the path for future research to fortify and refine these predictive models, with the ultimate aim of enhancing cancer care and management across diverse populations.

## Figures and Tables

**Table 1:** Selected baseline characteristics by stage of diagnosis status.

Patient Characteristics	Early *n* = 17,884 (42.74%)	Late *n* = 23,955 (57.26%)	
	Frequency (%) or Mean (SE)	Frequency (%) or Mean (SE)	*p*-Value
Age at Diagnosis			<0.0001
Age in years, Mean (SE)	66.72 (0.1)	65.85 (0.1)	
Age Group			<0.0001
18–39	471 (2.63%)	790 (3.30%)	
40–49	1262 (7.06%)	2288 (9.55%)	
50–64	5678 (31.75%)	7578 (31.63%)	
65+	10,473 (58.56%)	13,299 (55.52%)	
Race & Ethnicity			0.005
NH White	12,263 (68.67%)	16,305 (68.19%)	
NH Black	3263 (18.27%)	4279 (17.89%)	
Hispanic	1786 (10.00%)	2517 (10.53%)	
NH Asian	440 (2.46%)	702 (2.94%)	
NH Pacific Islander	68 (0.38%)	74 (0.31%)	
Unknown/Not Documented	39 (0.22%)	35 (0.15%)	
Marital Status at Diagnosis			<0.0001
Married	9578 (57.00%)	11,935 (53.95%)	
Unmarried	6523 (38.82%)	9437 (42.66%)	
Unknown	702 (4.18%)	752 (3.40%)	
Primary Payer at Diagnosis			<0.0001
Medicaid	284 (1.70%)	636 (2.83%)	
Medicare	9267 (55.52%)	11,762 (52.28%)	
Private Insurance	6124 (36.69%)	8295 (36.87%)	
Military	601 (3.60%)	680 (3.02%)	
Indian/Public Health Service	2 (0.01%)	1 (0.00%)	
Self-pay/Uninsured	413 (2.47%)	1122 (4.99%)	
Year of Diagnosis			<0.0001
2000–2004	5346 (29.89%)	7242 (30.23%)	
2005–2009	4836 (27.04%)	5882 (24.55%)	
2010–2014	3763 (21.04%)	4917 (20.53%)	
2015–2019	3821 (21.37%)	5705 (23.82%)	
2020	118 (0.66%)	209 (0.87%)	
Stage of disease at diagnosis			<0.0001
In Situ	2594 (14.50%)	0 (0.00%)	
Localized	15,290 (85.50%)	0 (0.00%)	
Regional	0 (0.00%)	16,005 (66.81%)	
Distant	0 (0.00%)	7950 (33.19%)	
Not Staged/Unknown	0 (0.00%)	0 (0.00%)	
Primary Site			<0.0001
Colon	12,521 (70.01%)	17,645 (73.66%)	
Rectum	5363 (29.99%)	6310 (26.34%)	
Grade			<0.0001
Grade I	3421 (19.13%)	2390 (9.98%)	
Grade II	8589 (48.03%)	13,067 (54.55%)	
Grade III	1069 (5.98%)	4162 (17.37%)	
Grade IV	194 (1.08%)	570 (2.38%)	
T-cell	1 (0.01%)	1 (0.00%)	
B-cell	89 (0.50%)	92 (0.38%)	
NK Cell	1 (0.01%)	0 (0.00%)	
Unknown Grade	4520 (25.27%)	3673 (15.33%)	
Treatment Status			<0.0001
No treatment given	351 (4.54%)	711 (6.54%)	
Treatment given	7256 (93.88%)	10,044 (92.44%)	
Active surveillance (watchful waiting)	17 (0.22%)	5 (0.05%)	
Unknown if treatment was given	105 (1.36%)	106 (0.98%)	
Days between date of initial diagnosis and date first course of treatment			0.2142
Time Lag, Mean (SE)	9.93 (1.97)	13.67 (2.28)	
Surgery			<0.0001
Yes	16,627 (92.97%)	19,698 (82.23%)	
No	1077 (6.02%)	4060 (16.95%)	
Unknown	180 (1.01%)	197 (0.82%)	
Chemotherapy			<0.0001
Yes	1835 (10.26%)	13,276 (55.42%)	
No	15,575 (87.09%)	10,330 (43.12%)	
Unknown	474 (2.65%)	349 (1.46%)	
Radiation Therapy			<0.0001
Yes	1384 (7.75%)	4063 (16.97%)	
No	15,541 (86.98%)	18,717 (78.19%)	
Unknown	943 (5.28%)	1157 (4.83%)	
Vital Status			<0.0001
Dead	7275 (40.68%)	14,859 (62.03%)	
Alive	10,609 (59.32%)	9096 (37.97%)	

**Table 2:** Selected neighborhood census tracts characteristics by stage of diagnosis status.

Census Tract Characteristics	Early *n* = 17,884 (42.74%)	Late *n* = 23,955 (57.26%)	
	Mean (SE)	Mean (SE)	*p*-Value
Access to Healthcare			
Percent Uninsured	7.816 (0.04)	7.9552 (0.04)	0.0085
Doctor Checkup in Past Year Among Adults (2020)	76.7273 (2.98)	76.5405 (3.03)	<0.0001
Doctor Checkup in Past Year Among Adults (2020)	76.7273 (2.98)	76.5405 (3.03)	<0.0001
Population with a Disability (2017–2021)	13.2833 (0.05)	13.1551 (0.04)	0.0372
Socio Economic			
% Spent on Housing & Transportation	53.5814 (0.06)	53.2928 (0.05)	0.0003
%Spent on Housing	26.0618 (0.01)	26.0798 (0.01)	0.2533
% Spent on Transportation	27.5196 (0.06)	27.213 (0.06)	0.0003
Employment Access Index (2016)	20,613.6 (177)	21,241.2 (158)	0.0082
Labor Force Participation Rate (2017–2021)	63.3817 (0.08)	63.6564 (0.07)	0.0093
Median Household Income (2017–2021)	82,806.2 (326.1)	83,742.1 (285.2)	0.0311
% Pop with Access to Healthy Food	0.7214 (0.002)	0.7144 (0.001)	0.0088
Educational Attainment			
Less than High school education	9.8888 (0.05)	10.027 (0.05)	0.0446
Educational Attainment—Some College No Degree (2017–2021)	8.1371 (0.03)	8.0137 (0.02)	0.0005
Behaviors			
Binge Drinking Among Adults (2020)	15.3011 (0.02)	15.3652 (0.01)	0.0017
Race and Ethnicity			
White per capita	0.633 (0.002)	0.6335 (0.002)	0.8212
Black per capita	0.2003 (0.002)	0.1952 (0.001)	0.0141
Asian per capita	0.0483 (0.006)	0.0495 (0.005)	0.0987
Hispanic per capita	0.0789 (0)	0.0821 (0)	0.0008
Environmental Exposures			
Air Quality: Respiratory Hazard Index (2014)	0.4039 (0.005)	0.4051 (0.004)	0.0707
Diesel Particulate Matter Environmental Justice Index (2021)	14.1463 (0.1)	14.398 (0.09)	0.0501
Diesel Particulate Matter Level in Air (2021)	0.2206 (0.008)	0.224 (0.007)	0.0026
Underground Storage Tanks (2021)	5.044 (0.05)	5.2027 (0.04)	0.0114
Population Weighted Density	3359.9 (43.17)	3546.9 (39.87)	0.0015

**Table 3:** Results of machine learning model evaluation for late-stage diagnosis prediction.

Model	ROC-AUC	Overall Prediction Accuracy	Sensitivity	Specificity
Lasso (Penalized Logistic Regression)	0.7864	0.7159	0.7405	0.6975
Decision Tree	0.7006	0.7068	0.6580	0.7432
Random Forest	0.7554	0.7032	0.5640	0.8072
Gradient Boosting	0.8549	0.7725	0.7263	0.8070
SVM (SGD Classifier)	0.7298	0.6760	0.6793	0.6735

## Data Availability

Data are contained within the article and Supplementary Materials.

## Supplementary Materials

The following supporting information can be downloaded at: https://www.mdpi.com/article/10.3390/cancers16030540/s1, Table S1: Baseline Characteristics by Stage of Diagnosis Status; Table S2: Neighborhood Census Tracts Characteristics by Stage of Diagnosis Status.
