# Supplementary material for: Machine Learning as a Tool for Early Detection: A Focus on Late-Stage Colorectal Cancer across Socioeconomic Spectrums
Source: Cancers (Basel). 2024 Jan 26;16(3):540. doi: 10.3390/cancers16030540 (PMC10854986; doi:10.3390/cancers16030540)
Supplement: Supplementary file 1 [file cancers-16-00540-s001.zip › cancers-2825773-supplementary.pdf]

**Supplementary Materials**

**Table S1: Baseline Characteristics by Stage of Diagnosis Status**

| **Patient Characteristics** | **Early N = 17884 (42.74%)** | **Late N = 23955 (57.26%)** | |
|---|---|---|---|
| | **Frequency (%) or Mean (SE)** | **Frequency (%) or Mean (SE)** | **P-value** |
| **Age at Diagnosis** | | | **<.0001** |
| Age in years, Mean (SE) | 66.72 (0.1) | 65.85 (0.1) | |
| **Age Group** | | | **<.0001** |
| 18-39 | 471 (2.63%) | 790 (3.30%) | |
| 40-49 | 1262 (7.06%) | 2288 (9.55%) | |
| 50-64 | 5678 (31.75%) | 7578 (31.63%) | |
| 65+ | 10473 (58.56%) | 13299 (55.52%) | |
| **Race & Ethnicity** | | | **0.005** |
| NH White | 12263 (68.67%) | 16305 (68.19%) | |
| NH Black | 3263 (18.27%) | 4279 (17.89%) | |
| Hispanic | 1786 (10.00%) | 2517 (10.53%) | |
| NH Asian | 440 (2.46%) | 702 (2.94%) | |
| NH Pacific Islander | 68 (0.38%) | 74 (0.31%) | |
| Unknown/Not Documented | 39 (0.22%) | 35 (0.15%) | |
| **Ethnicity** | | | **0.0188** |
| Non-Spanish/Non-Hispanic | 16095 (90.00%) | 21436 (89.48%) | |
| Unknown whether Spanish or not | 1518 (8.49%) | 2072 (8.65%) | |
| Spanish/Hispanic | 271 (1.52%) | 447 (1.87%) | |
| **Marital Status at Diagnosis** | | | **<.0001** |
| Married | 9578 (57.00%) | 11935 (53.95%) | |
| Unmarried | 6523 (38.82%) | 9437 (42.66%) | |
| Unknown | 702 (4.18%) | 752 (3.40%) | |
| **Primary Payer at Diagnosis** | | | **<.0001** |
| Medicaid | 284 (1.70%) | 636 (2.83%) | |
| Medicare | 9267 (55.52%) | 11762 (52.28%) | |
| Private Insurance | 6124 (36.69%) | 8295 (36.87%) | |
| Military | 601 (3.60%) | 680 (3.02%) | |
| Indian/Public Health Service | 2 (0.01%) | 1 (0.00%) | |
| Self-pay/Uninsured | 413 (2.47%) | 1122 (4.99%) | |
| **Alcohol History** | | | **0.5686** |

| Patient Characteristics | Early<br>N = 17884<br>(42.74%) | Late<br>N = 23955<br>(57.26%) | |
|---|---|---|---|
| Current user | 0 (0.00%) | 0 (0.00%) | |
| Never used | 6 (75.00%) | 3 (60.00%) | |
| Unknown | 2 (25.00%) | 2 (40.00%) | |
| **Tobacco History** | | | **0.6271** |
| Current user | 2 (22.22%) | 2 (40.00%) | |
| Never used | 4 (44.44%) | 1 (20.00%) | |
| Previous user | 0 (0.00%) | 0 (0.00%) | |
| Unknown | 3 (33.33%) | 2 (40.00%) | |
| **Year of Diagnosis** | | | **<.0001** |
| 2000-2004 | 5346 (29.89%) | 7242 (30.23%) | |
| 2005-2009 | 4836 (27.04%) | 5882 (24.55%) | |
| 2010-2014 | 3763 (21.04%) | 4917 (20.53%) | |
| 2015-2019 | 3821 (21.37%) | 5705 (23.82%) | |
| 2020 | 118 (0.66%) | 209 (0.87%) | |
| **Stage of disease at diagnosis** | | | **<.0001** |
| In Situ | 2594 (14.50%) | 0 (0.00%) | |
| Localized | 15290 (85.50%) | 0 (0.00%) | |
| Regional | 0 (0.00%) | 16005 (66.81%) | |
| Distant | 0 (0.00%) | 7950 (33.19%) | |
| Not Staged/Unknown | 0 (0.00%) | 0 (0.00%) | |
| **Primary Site** | | | **<.0001** |
| Colon | 12521 (70.01%) | 17645 (73.66%) | |
| Rectum | 5363 (29.99%) | 6310 (26.34%) | |
| **Grade** | | | **<.0001** |
| Grade I | 3421 (19.13%) | 2390 (9.98%) | |
| Grade II | 8589 (48.03%) | 13067 (54.55%) | |
| Grade III | 1069 (5.98%) | 4162 (17.37%) | |
| Grade IV | 194 (1.08%) | 570 (2.38%) | |
| T-cell | 1 (0.01%) | 1 (0.00%) | |
| B-cell | 89 (0.50%) | 92 (0.38%) | |
| NK Cell | 1 (0.01%) | 0 (0.00%) | |
| Unknown Grade | 4520 (25.27%) | 3673 (15.33%) | |
| **Laterality at Diagnosis** | | | **0.0039** |
| Not a paired site | 17756 (99.28%) | 23754 (99.16%) | |
| Right: origin of primary | 108 (0.60%) | 151 (0.63%) | |

| Patient Characteristics | Early<br>N = 17884<br>(42.74%) | Late<br>N = 23955<br>(57.26%) | |
|---|---|---|---|
| Left: origin of primary | 20 (0.11%) | 33 (0.14%) | |
| Only one side involved; right or left origin unspecified | 0 (0.00%) | 0 (0.00%) | |
| Bilateral | 0 (0.00%) | 0 (0.00%) | |
| Unknown laterality | 0 (0.00%) | 17 (0.07%) | |
| **Tumor Size (cm)** | | | **<.0001** |
| No mass/tumor found | 1 (0.04%) | 6 (0.15%) | |
| 0.1 cm or less than 0.1 cm | 26 (0.99%) | 2 (0.05%) | |
| 0.2 cm to 98.8 cm | 1963 (74.90%) | 2989 (73.86%) | |
| 98.9 cm or larger | 0 (0.00%) | 3 (0.07%) | |
| Familial/Multiple polyposis | 3 (0.11%) | 1 (0.02%) | |
| Microscopic focus only | 6 (0.23%) | 3 (0.07%) | |
| Unknown; size not stated | 622 (23.73%) | 1043 (25.77%) | |
| **Treatment Status** | | | **<.0001** |
| No treatment given | 351 (4.54%) | 711 (6.54%) | |
| Treatment given | 7256 (93.88%) | 10044 (92.44%) | |
| Active surveillance (watchful waiting) | 17 (0.22%) | 5 (0.05%) | |
| Unknown if treatment was given | 105 (1.36%) | 106 (0.98%) | |
| **Days between date of initial diagnosis and date first course of treatment** | | | **0.2142** |
| Time Lag, Mean (SE) | 9.93 (1.97) | 13.67 (2.28) | |
| **Surgery** | | | **<.0001** |
| Yes | 16627 (92.97%) | 19698 (82.23%) | |
| No | 1077 (6.02%) | 4060 (16.95%) | |
| Unknown | 180 (1.01%) | 197 (0.82%) | |
| **Chemotherapy** | | | **<.0001** |
| Yes | 1835 (10.26%) | 13276 (55.42%) | |
| No | 15575 (87.09%) | 10330 (43.12%) | |
| Unknown | 474 (2.65%) | 349 (1.46%) | |
| **Radiation Therapy** | | | **<.0001** |
| Yes | 1384 (7.75%) | 4063 (16.97%) | |
| No | 15541 (86.98%) | 18717 (78.19%) | |
| Unknown | 943 (5.28%) | 1157 (4.83%) | |

| Patient Characteristics | Early<br>N = 17884<br>(42.74%) | Late<br>N = 23955<br>(57.26%) | |
|---|---|---|---|
| **Hormone Therapy** | | | **<.0001** |
| Yes | 23 (0.13%) | 75 (0.31%) | |
| No | 17470 (97.69%) | 23488 (98.05%) | |
| Unknown | 391 (2.19%) | 392 (1.64%) | |
| **Vital Status** | | | **<.0001** |
| Dead | 7275 (40.68%) | 14859 (62.03%) | |
| Alive | 10609 (59.32%) | 9096 (37.97%) | |
| **Cancer Status** | | | **<.0001** |
| No evidence of this tumor | 13295 (74.98%) | 9802 (41.16%) | |
| Evidence of this tumor | 2069 (11.67%) | 10937 (45.92%) | |
| Unknown | 2367 (13.35%) | 3077 (12.92%) | |

**Table S2: Neighborhood Census Tracts Characteristics by Stage of Diagnosis Status**

| Census Tract Characteristics | Early<br>N = 17884<br>(42.74%) | Late<br>N = 23955<br>(57.26%) | |
|---|---|---|---|
| | **Mean (SE)** | **Mean (SE)** | **P-value** |
| Access to Healthcare | | | |
| Total population | 4282.4 (11.52) | 4305.2 (10.13) | 0.138 |
| Percent Uninsured | 7.816 (0.04) | 7.9552 (0.04) | 0.0085 |
| Doctor Checkup in Past Year Among Adults (2020) | 76.7273 (2.98) | 76.5405 (3.03) | <.0001 |
| Fair or Poor General Health Among Adults (2020) | 14.9915 (0.04) | 14.9312 (0.04) | 0.2699 |
| Primary Care Nurse Practitioners to Pop Ratio (2022) | 74.1199 (1.53) | 73.7814 (1.3) | 0.8664 |
| Primary Care Physicians to Population Ratio (2022) | 96.8316 (2.16) | 97.8913 (1.87) | 0.7108 |

| Census Tract Characteristics | Early<br>N = 17884<br>(42.74%) | Late<br>N = 23955<br>(57.26%) | |
|---|---|---|---|
| Population with a Disability (2017-2021) | 13.2833 (0.05) | 13.1551 (0.04) | 0.0372 |
| Socio Economic | | | |
| % Spent on Housing & Transportation | 53.5814 (0.06) | 53.2928 (0.05) | 0.0003 |
| %Spent on Housing | 26.0618 (0.01) | 26.0798 (0.01) | 0.2533 |
| % Spent on Transportation | 27.5196 (0.06) | 27.213 (0.06) | 0.0003 |
| Employment Access Index (2016) | 20613.6 (177) | 21241.2 (158) | 0.0082 |
| Labor Force Participation Rate (2017-2021) | 63.3817 (0.08) | 63.6564 (0.07) | 0.0093 |
| Total Households Below Poverty Level per household (2017-2021) | 0.1058 (0.006) | 0.1054 (0.005) | 0.6361 |
| Median Household Income (2017-2021) | 82806.2 (326.1) | 83742.1 (285.2) | 0.0311 |
| Income Inequality (Gini Index) (2017-2021) | 0.4098 (0.005) | 0.4095 (0.005) | 0.5667 |
| Townsend Index | 0.2538 (0.008) | 0.2552 (0.007) | 0.2313 |
| Educational Attainment | | | |
| Less than High school education | 9.8888 (0.05) | 10.027 (0.05) | 0.0446 |
| Educational Attainment - High School Degree (2017-2021) | 26.0016 (0.09) | 26.0476 (0.08) | 0.6975 |
| Educational Attainment - Some College No Degree (2017-2021) | 8.1371 (0.03) | 8.0137 (0.02) | 0.0005 |
| Educational Attainment - Associates Degree (2017-2021) | 19.3612 (0.05) | 19.2403 (0.04) | 0.0612 |
| Educational Attainment - Bachelor's Degree (2017-2021) | 20.8526 (0.08) | 20.7858 (0.07) | 0.5051 |
| Educational Attainment - Graduate Degree (2017-2021) | 15.7586 (0.09) | 15.8857 (0.08) | 0.305 |
| Behaviors | | | |
| Regular Smoking Among Adults (2020) | 16.6818 (0.04) | 16.6744 (0.03) | 0.8865 |
| No Leisure-Time Physical Activity Among Adults (2020) | 22.6601 (0.05) | 22.6251 (0.04) | 0.5683 |

| Census Tract Characteristics | Early<br>N = 17884<br>(42.74%) | Late<br>N = 23955<br>(57.26%) | |
|---|---|---|---|
| Binge Drinking Among Adults (2020) | 15.3011 (0.02) | 15.3652 (0.01) | 0.0017 |
| Race and Ethnicity | | | |
| White per capita | 0.633 (0.002) | 0.6335 (0.002) | 0.8212 |
| Black per capita | 0.2003 (0.002) | 0.1952 (0.001) | 0.0141 |
| Asian per capita | 0.0483 (0.006) | 0.0495 (0.005) | 0.0987 |
| Hawaiian per capita | 0.000458 (0.0002) | 0.000447 (0.0001) | 0.6473 |
| Hispanic per capita | 0.0789 (0) | 0.0821 (0) | 0.0008 |
| American Indian per capita | 0.0016 (0.004) | 0.0016 (0.006) | 0.8839 |
| Single Race Other per capita | 0.00356 (0.006) | 0.00355 (0.005) | 0.8906 |
| Two Or More Races Other per capita | 0.0339 (0.002) | 0.0342 (0.002) | 0.4153 |
| Racial Diversity Index | 0.8518 (0.003) | 0.85 (0.002) | 0.5966 |
| Environmental Exposures | | | |
| Air Quality: Respiratory Hazard Index (2014) | 0.4039 (0.005) | 0.4051 (0.004) | 0.0707 |
| Air Quality: Individual Lifetime Cancer Risk (2014) | 30.309 (0.03) | 30.3504 (0.03) | 0.331 |
| Proximity to Treatment Storage and Disposal Facilities (2021) | 0.6214 (0.01) | 0.6269 (0.01) | 0.5636 |
| Proximity to Treatment Storage and Disposal Facilities Environment | 12.1676 (0.09) | 12.1421 (0.08) | 0.8354 |
| Proximity to Risk Management Plan Sites Environmental Justice Index | 12.5844 (0.1) | 12.501 (0.09) | 0.5432 |
| Proximity to Risk Management Plan Sites (2021) | 0.3914 (0.004) | 0.3927 (0.004) | 0.8200 |
| Respiratory Hazard Environmental Justice Index (2021) | 20.8894 (0.11) | 20.9536 (0.09) | 0.6466 |
| Proximity to National Priorities List Sites (2021) | 0.1002 (0.002) | 0.1036 (0.001) | 0.1129 |

| Census Tract Characteristics | Early N = 17884 (42.74%) | Late N = 23955 (57.26%) | |
|---|---|---|---|
| Lead Paint Environmental Justice Index (2021) | 13.7564 (0.09) | 13.7142 (0.08) | 0.7311 |
| Diesel Particulate Matter Environmental Justice Index (2021) | 14.1463 (0.1) | 14.398 (0.09) | 0.0501 |
| Diesel Particulate Matter Level in Air (2021) | 0.2206 (0.008) | 0.224 (0.007) | 0.0026 |
| Underground Storage Tanks (2021) | 5.044 (0.05) | 5.2027 (0.04) | 0.0114 |
| Proximity to National Priorities List Sites Environmental Justice | 15.1285 (0.1) | 15.3434 (0.09) | 0.1149 |
| Air Toxics Cancer Risk Environmental Justice Index (2021) | 24.8407 (0.12) | 24.708 (0.1) | 0.3979 |
| Underground Storage Tanks Environmental Justice Index (2021) | 14.9117 (0.09) | 15.0342 (0.08) | 0.3233 |
| Particulate Matter Environmental Justice Index (2021) | 7.502 (0.05) | 7.5896 (0.04) | 0.1439 |
| Ozone Environmental Justice Index (2021) | 10.2882 (0.05) | 10.2932 (0.04) | 0.9376 |
| Proximity to Major Direct Water Dischargers Environmental Justice | 8.7443 (0.08) | 8.6011 (0.07) | 0.1546 |
| Proximity to Major Direct Water Dischargers (2021) | 3.2877 (0.68) | 2.9857 (0.5) | 0.7220 |
| Traffic Proximity and Volume (2021) | 528.9 (7.23) | 546.5 (6.44) | 0.0698 |
| Traffic Proximity and Volume Environmental Justice Index (2021) | 14.9135 (0.11) | 15.0484 (0.1) | 0.3485 |
| Population Weighted Density | 3359.9 (43.17) | 3546.9 (39.87) | 0.0015 |
| Walkability Index | 11.4083 (0.03) | 11.4006 (0.03) | 0.8610 |
| Extra Variables | | | |
| Incarceration rate per 100,000 | 587.5 (4.35) | 583 (3.78) | 0.4315 |
| % Pop with Access to Healthy Food | 0.7214 (0.002) | 0.7144 (0.001) | 0.0088 |
| Diagnosed Depression Among Adults (2020) | 19.4715 (0.02) | 19.4478 (0.01) | 0.2791 |
